# A scoping review of mentorship of health personnel to improve the quality of health care in low and middle-income countries

**DOI:** 10.1186/s12992-017-0301-1

**Published:** 2017-10-03

**Authors:** Patricia Schwerdtle, Julia Morphet, Helen Hall

**Affiliations:** 1 Nursing & Midwifery, Faculty of Medicine, Nursing and Health Sciences, Monash University, Clayton, VIC Australia; 2 Monash Emergency Research Collaborative, Nursing & Midwifery, Monash Health, Clayton, Dandenong Australia; 3 Medecins Sans Frontieres, Sydney, NSW Australia

**Keywords:** Mentorship, Health personnel, LMICs, Quality of care, Scoping review

## Abstract

**Background:**

Most Low and Middle-Income Countries are facing a crisis in human resources for health which compromises their ability to meet health related targets outlined by the Sustainable Development Goals. The crisis is not limited to the availability of health personnel but also the quality of care and the training and development of the workforce. To address these challenges, evidence based education strategies are urgently required. Mentorship has been found to improve health personnel performance in High-Income Countries however, little is known about its role in Low and Middle-Income Countries. To address this gap in understanding, we conducted a scoping review of the current literature.

**Methods:**

CINAHL, EMBASE and OVID Medline were systematically searched along with grey literature for peer-reviewed research papers specific to the research question. A six-step scoping review framework was utilised to identify the relevant literature and summarise the pertinent findings.

**Results:**

The initial search identified 592 records, and five papers, reporting on four studies, were retained for data charting and extraction. All four studies described a positive effect of mentorship on the quality of care outcomes. The results are collated according to features of the intervention including mentor training, mentor-mentee ratios, mentorship model, intervention intensity and key findings in terms of outcome measures.

**Conclusions:**

This review identifies a paucity of evidence of mentorship in this context however, current evidence supports the assertion that effective mentorship contributes to the improvement of certain quality of care outcomes. The features of successful mentorship interventions are outlined and the implications are discussed in the context of existing evidence.

## Background

Low and Middle-Income Countries (LMICs), also known as developing countries, are defined as nations with a lower standard of living, under-developed industry, and a low human development index compared to other countries [1]. LMICs carry a disproportionately high burden of disease compared to High-Income Countries (HICs) yet, the vast majority of health workers live in HICs, while LMICs are challenged by a severe shortage of health personnel. This shortage is further complicated by a maldistribution of staff, inadequate training, a lack of development opportunities, skill mix imbalance, high patient ratios, increasingly complicated medical programs and limited physical resources, which are further compromised by challenging socio-cultural-political-economic environments [2, 3].

There is a broad consensus among policy makers, researchers and practitioners that accessible, qualified and responsive Human Resources for Health (HRH) are a critical determinant of the health of populations [4]. Apart from other factors, such as systems, supplies and equipment, the quality of care delivered by appropriately prepared health personnel is central to improving health outcomes in LMICs [5]. HRH underlies quality and service delivery improvements and increased investment towards health professional education is crucial in order to achieve universal coverage [6].

There is growing concern of a widespread health worker crisis, especially in resource-poor settings [7]. In the context of a grossly mal-distributed healthcare workforce globally, many low income countries have serious difficulty producing, recruiting and retaining health professionals [2]. Health personnel in developing countries are challenged by limited opportunities for formal training, high turnover and under-prioritized continuing education. When education for health care providers does occur, it often takes the form of large centralized, didactic trainings at district or capital levels, prioritizing senior and management staff incentivized with per diems [8–10]. This model may marginalize and disadvantage rural health staff in a primary health care model that is largely decentralized.

LMICs have a strong history of practical, directed learning which has been the basis of education in many health disciplines. Over the past decade however, there has been a noticeable move towards theoretical and evidence-based practice in developing countries in order to modernize teaching methods and improve the quality of care [11]. Mentorship may be an innovative strategy to address the quality of care issues and supersede traditional supervision techniques which are often top-down, compliance-centred and can be disempowering.

Mentorship is a flexible teaching and learning process that serves specific objectives of the health worker and health care service [12]. This approach is relationship oriented and designed to develop the professional capacity of both parties. Mentorship is based on mutual trust and respect, it seeks to build confidence and is an empowering partnership between two people who have a shared set of learning objectives [13]. The term mentoring is sometimes confused with clinical teaching or coaching. Clinical teaching occurs when a student engages with a clinician who assumes responsibility for patient care and student learning [14]. While coaching, is a method of directing, instructing and training a person usually with the aim of developing specific skills in that individual [15].

**Fig. 1:**
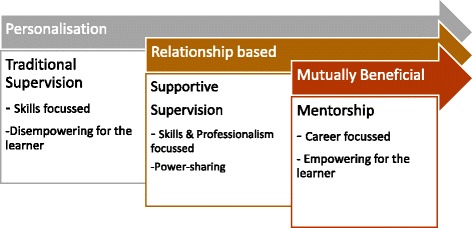
Diagrammatic Representation of Supervision and Mentorship

Figure 1 provides a diagrammatic representation of traditional supervision, supportive supervision and mentorship, outlining some of the key distinguishing features. When supervision moves to a mentorship model, it becomes more personalised, relationship-based, and mutually beneficial. Mentorship shifts the focus of the intervention from individual skills, often focussing on a specific service or health issue (e.g. immunisation or hand hygiene) to a holistic, learner-based, professional and career focus. Power is transferred from the supervisor to a shared-power model, whereby the learner can identify their own learning needs and identify systems and process issues that may also negatively impact the quality of care.

There is only limited research exploring the role of mentorship in LMICs. A recent systematic review revealed that mentorship has been implemented to strengthen leadership and management competencies in LMICs however, it did not focus on clinical practice [12]. Another recent scoping review investigated a range of approaches to support and improve the performance of primary health care workers in LMICs [16]. While the review provided valuable insights, it included four approaches other than mentorship, including supervision, quality improvement interventions, tools, aids and technology and coaching [16]. The aim of this scoping review is to explore the literature published from 2007 onwards, investigating the role of mentorship of health personnel to improve the quality of care, defined broadly as adherence to an accepted standard or guideline, in LMICs.

## Methods

This scoping review was conducted according to the steps outlined by Arksey & O’Malley [17]. This review was triggered by an expert consultation with a leading international medical humanitarian aid organization. The organization proposed mentorship as a strategy to develop the knowledge requirements of a field intervention aimed at improving the quality of care in primary health care settings in developing countries.

In order to explore and systematically map the literature examining this topic, a scoping methodology was chosen [18]. Scoping reviews aim to determine the extent of existing evidence and identify gaps requiring further study, or to inform a subsequent systematic review, possibly leading to an evidence based intervention that can be tested in the field. A scoping methodology was considered appropriate because the feasibility of conducting a systematic review on mentorship in LMICs was challenging due to a paucity of relevant literature [17, 19].

This review specifically aimed to address the following questions:

- What is known about mentorship of health personnel as a strategy to improve the quality of care in LMICs?
- What are the key findings and recommendations of existing research on this topic?
- Where do the research gaps lie?

### Search strategy

A three stage search strategy was conducted, starting with an initial limited search of MEDLINE and CINAHL, followed by an analysis of keywords contained in the title and abstract of retrieved papers. A second search was then undertaken in OVID Medline, CINAHL and EMBASE using all identified keywords and index terms. Thirdly, the reference lists of all selected articles were searched for articles that met the selection criteria. In addition, a grey literature search was conducted using the same keywords.

### Search terms

The search strategy, developed with a research librarian, included the keywords: ‘health personnel’, OR ‘nurse’, OR ‘community health worker’, OR ‘medical staff’, OR ‘doctor’, OR ‘physicians’ AND ‘mentor*’, OR ‘preceptor*’, OR ‘buddy*’, OR ‘supervis*’ AND ‘developing countr*’, OR ‘third world countr*’, OR ‘low income countr*’, OR ‘middle-income countr*’, OR ‘resource constrained setting’.

### Inclusion and exclusion criteria

Studies that described health personnel as the population were included. This review focused on health personnel who delivered healthcare in a primary health care context. For the purposes of this review this included nurses, community health workers, medical staff, doctors, physicians, rural health personnel, physician assistants, field workers and clinical officers. As the study focussed on LMICs, field worker was added as a keyword to include health personnel working in humanitarian and conflict settings. Students, pharmacists and allied health professionals were excluded from the study.

Studies were included if the mentorship program aimed to improve the quality of care. Programs, initiatives and research related to management systems, policy, leadership, partnerships and finance were excluded. Studies describing clinical education, coaching or supervision, which was different from the operational definition of mentorship, were excluded.

This review was limited to papers published in English that reported research. Opinion pieces, letters to the editor, symposia proceedings and press releases were excluded.

Literature published from 2007 onwards was included to ensure contemporary practice in LMICs was captured. This date was considered appropriate because in 2006 the World Health Organization (WHO) declared a ‘global health workforce crisis’, which led to increased funding for health worker training and development in LMICs [20].

### Study selection

Studies retrieved from the searches were screened by two independent reviewers using a predetermined template. Titles and abstracts were initially screened against the inclusion criteria, then full texts were screened for eligibility by the same reviewers. Disagreements were discussed with a third reviewer until consensus was achieved. If further information was required for selection, attempts were made to contact the authors of the primary studies.

## Results

**Fig. 2:**
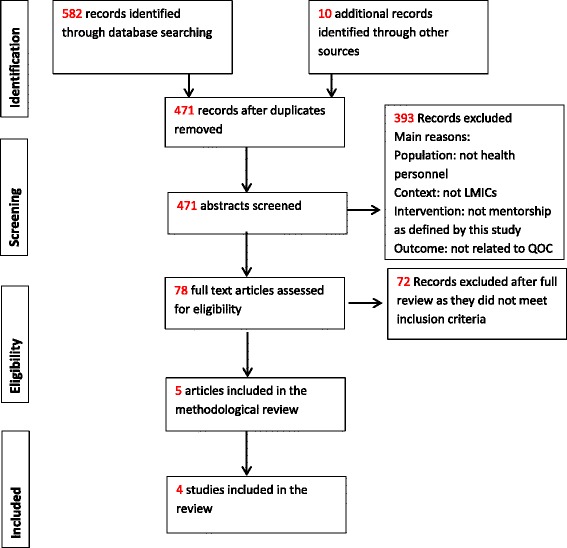
PRISMA Flow Chart; Scoping Review Results

The systematic search of three electronic databases and grey literature returned a total of 592 articles. After duplicates were removed, 471 studies remained. Following review of title and abstract, 78 articles remained for full-text review. Of these 78 articles, four studies (five papers) met the inclusion criteria (See Fig. 2: PRISMA flowchart).

The main reasons for article exclusion were that the population studied were not health personnel, the context of the study was not LMICs, the intervention did not meet the operational definition of mentorship as outlined by the search strategy or that the outcome measure was not related to the quality of care.

### Characteristics of mentorship programs in LMIC

The five papers included in this study, examined the quality of care outcomes secondary to mentorship interventions in Rwanda [8, 21], Afghanistan [22], Jordan [23] and Botswana [24]. Articles written by Anatole et al. [8] and Magge et al. [21] reported on different aspects of the same study. In two studies, mentors from developed countries worked with mentees from developing countries [22, 23], whilst in the remaining studies, mentors and mentees were from the same country [8, 21, 24]. The important characteristics of the studies included in this review are outlined in Table 1.

**Table 1:** Data Charting - Summary of selected studies addressing the research question

Author	Country of study	Study Aim	Study Design	Population (Mentor/Mentee)	Intervention	Program duration	Frequency of contact	Outcome Measures	Key Findings
Anatole et al. (2013) [8]	Rwanda	Interim evaluation of a nursing mentorship program	Observational checklists completed by mentors, retrospective review of patient records	Rwandan Nurses/Rwandan Nurses	Mentoring and Enhanced Supervision at Health Centres (MESH), ‘Side by side’ mentorship. Mentors received extra training and were integrated into the hospital team	Ongoing	2–3 days/month	Number & accuracy of Integrated Management of Childhood Illness patient assessments	‘Side by side’ mentorship improved the quality of care outcomes, and is feasible and cost effective. Scale up was recommended. Significant increase in complete and accurate patient assessments.
Beckett et al. (2015) [22]	Afghanistan	To evaluate a mentorship intervention by exploring mentee experience. To improve pre-deployment training of mentors	Cross-sectional survey of mentees	Canadian military physicians & surgeons/Afghan physicians, nurses and health personnel	‘Team to team’ using CANMEDS mentoring techniques	12 months	Not reported	Mentees perceptions of training, feedback, communication & availability of mentors	Most mentees found mentorship a positive experience. Mentees criticised mentors for failing to consider features of a low-resource setting.
Finley et al. (2008) [23]	Jordan	To describe a capacity building program incorporating mentorship to develop, implement and evaluate a paediatric pain management program.	Interviews, focus groups, field observation, retrospective review of patient records	Canadian Clinical Nurse Specialists & Anaesthetist/Jordanian Nurses and Oncologists	‘Team to team mentorship’ Implementing a paediatric pain management service involving mentorship	24 months	3 visits through-out the program	Documented pain assessments, Administration of opioid analgesia;	Increased analgesia administration following the program. Mentorship was considered an important factor in the effectiveness of an intervention involving a High-Income Country – Low Income Country partnership.
Magge et al. (2015) [24]	Rwanda	To measure the change in the quality of care following the addition of a mentorship intervention to didactic training	Pre-post intervention study	MOH Nurse/Health Centre Nurses	‘Side by side’ model including clinical coaching through case observation, case based and didactic teaching, feedback of performance data and QI facilitation.	Ongoing	‘regular visits’	Adherence to Integrated Management of Childhood Illness (IMCI) Assessment Index Number of children seen	Significant improvement in IMCI assessment, classification and treatment, improvement in percentage of children given correct treatment, improved IMCI coverage (proportion of children seen).
Workneh et al. (2012) [21]	Botswana	To examine the quality of care pre and post introduction of mentoring program	Retrospective patient chart review	Botswana experienced medical officers & nurses/Botswana medical officers & nurses	‘Side by side’ mentoring during patient care	Ongoing	1/month	Completion of documentation (viral load count, patient education), pill counts (indicates patients taking medications as prescribed), antiretroviral dosing, lab monitoring.	Improvement in the quality of care post-intervention. Specifically, significant increase in recording of viral load count, correct pill count, correct antiretroviral dosing, patient education documented, correct lab monitoring.

There was a range of professions represented in the populations studied: Nurses, physicians, surgeons, anaesthetists, oncologists and community health workers. The areas of practice in which mentorship occurred were primary health care [8], integrated management of childhood illness [21], field surgery [22], paediatric oncology [23] and paediatric HIV care [24].

Mentors were recruited in various ways including community nomination [21], a comprehensive multi-stage process based on national hiring procedures and WHO clinical mentoring guidelines [8], and a voluntary call from a pool of military physicians and surgeons [22].

Mentor preparation was diverse and usually limited to a few days. Preparation either ensured the mentor was already a content expert and focussed on training mentorship knowledge and skills [8, 22, 23], or incorporated both content and mentorship skills into mentor training [21]. One approach to mentorship training was a two-day clinical mentoring workshop, covering eight main areas; key principals of relationship building, communication skills and giving feedback, theories of adult learning, clinical teaching and mentoring techniques, applying clinical mentoring techniques, and using observation checklists for effective mentoring [8]. Beckett et al. [22] incorporated cultural sensitivity training into mentor preparation in addition to ‘train the trainer’ instruction and a competency framework was used to guide the three-day mentorship program.

The exact ratio of mentor to mentee was not always clearly described. Some studies allocated one mentor to each facility [8, 24], although mentors seemed to be ‘roaming’ and may have visited multiple centres during a rotation.

In terms of mentorship style, four studies described a ‘side by side’ model [8, 24, 21], which involved on-site mentorship focussing on relationship building, communication and feedback. In these studies, mentors and mentees were from the same cultural and professional background. Finley et al. [23] and Beckett et al. [22] described a ‘team to team’ mentorship model, and in addition to infrequent physical visits for role-modelling, Finley et al. [23] described the use of videoconferencing.

Intensity of the mentorship intervention could be categorised in terms of frequency and duration of visits, as well as the duration of the actual program. Visit frequency ranged from monthly [24] to six weekly [8] to annual visits interspersed with other contact forums [23]. Visit duration ranged from 1 day to an entire week where the mentor lived on-site and saw patients alongside mentees [8]. Two of the mentorship interventions (reported in 3 papers) appeared to be ongoing [8, 21, 24] whilst the other two mentorship programs lasted one year [22, 23].

### The Quality of care outcomes

All four studies reported in five papers recorded improvements in the quality of care as a result of the mentorship intervention [8, 21–24]. Outcome measures included more accurate documentation of care [8, 24], increased accordance between care plans and actual care delivered [21, 23, 24], and increased appropriate prescription and administration of analgesia [23] and antiretroviral drugs [24] (See Table 1).

Most researchers were supportive of mentorship [8, 21, 24], with one also reporting the intervention was cost-effective and should be scaled up [8]. Conversely, one study found that mentors from developed countries did not adjust their health care delivery style to match the limited resources of the LMIC setting. The mentees reported that the program was a positive experience, but highlighted a need for mentors to adjust their health care delivery expectations to match the resources available [22]. Finley et al. [23] found that while education played an important role in the improvement of the quality of care, policy change, role-modelling and mentorship were also important factors.

## Discussion

The aim of this review was to explore the current understanding of mentorship of health personnel as a strategy to improve the quality of care in LMICs. The review describes the key findings and recommendations based on existing evidence, whilst identifying where the research gaps lie. The review identified limited evidence on the use of mentorship in LMICs however, the studies did support the assertion that mentorship can improve the quality of care outcomes in this context. The features of successful mentorship interventions based on the identified studies are outlined below.

### Benefits of mentorship

The findings of this scoping review indicate that effective mentorship contributes to the improvement of certain quality of care outcomes in LMICs, which further supports the WHO recommendation stating mentorship is critical to sustain high quality clinical outcomes in resource constrained settings [25]. Furthermore, it is consistent with the findings of a systematic review examining mentorship in healthcare in high-income countries which concluded that it was an effective knowledge translation intervention [26]. Indeed, in high-income countries, clinical mentorship is considered an excellent training approach [27, 28]. While other reviews have found mentorship is integrated into management and leadership initiatives in LMICs [12], this review suggests it is less common, but may be beneficial as a clinical training and development strategy for health personnel.

### Features of a successful mentorship intervention

This review provided insight in terms of the optimal characteristics of mentorship interventions. Desirable features of a successful mentorship intervention include at least one dedicated mentor per facility, ensuring a mentor to mentee ratio where there is adequate staff and time to enable the mentor to feel well supported and to form meaningful relationships with mentees. Another desirable feature was adopting a supportive ‘side by side’ model where the mentor works alongside the mentee during the provision of care optimising opportunities for learning and the provision of constructive feedback. The review also found it desirable to prepare mentors appropriately, by not only ensuring discipline and context related knowledge and experience but also by developing skills such as relationship building and communication skills.

Another preferable feature identified in this review included ensuring congruency between the culture and discipline of the mentor and mentee. For example, Anatole et al. [8] and Magge et al. [24], who reported different outcomes of the same study, found that the mentoring of Rwandan nurses by Rwandan nurses was a strength of their intervention, as was the ongoing nature of the program. This aligns with McKenna & Stockhausen’s [15] definition of true mentorship in which the difference in the level of knowledge and experience between the mentor and mentee may be minimal and the duration of the arrangement long-term.

The findings from this review also highlight the need to consider the optimal intensity of the intervention which was described in terms of the frequency and duration of visits, and the duration of the entire program. This is clearly dependent on the context and available resources, however, the prominent intensity comprised of visits that lasted at least a few days and occurred at a minimum of monthly intervals and the most beneficial model was ongoing rather than discrete.

It is unclear whether mentorship is more suited to certain health programs, however in terms of studies identified by this review, mentorship seemed to be well suited to protocol driven primary health care areas such as Integrated Management of Childhood Illness (IMCI) and Maternal Child Health and HIV programs [21] which are settings where training and development opportunities are most scarce.

### Mentorship versus supervision

While mentorship is prevalent in healthcare in high-income countries [26], there is currently only limited literature reporting on evidence specifically for mentorship in LMICs**.** It is not clear if this is due to limited publication, however a significant volume of literature exploring supervision uncovered by the search strategy may indicate a dearth of mentorship as a training and development strategy in LMICs.

An alternative explanation for a lack of evidence was the possible confusion of terms. While some excluded studies used the word ‘mentorship’, the description of the intervention matched supervision, rather than the operational definition of mentorship used in this study. These studies often involved a high number of participants, with only a few supervisors, and concentrated on skill development, using checklists to evaluate compliance to guidelines. They were often limited in intensity and duration and did not focus on the development and continuation of a professional relationship that benefitted both the mentor and mentee. The mentee benefits from mentorship through career advancement, learning, development and feedback, while the mentor may benefit through a positive impact on their practice, personal fulfilment, increased confidence, new ideas and revitilised interest in their work [29].

In the last decade, traditional supervision, which is characterised by a hierarchical structure, has been replaced with a more supportive form of supervision where communication is enhanced and decision making is shared. Supportive supervision has an ongoing practical focus integrating constructive feedback, rather than judging performance based on predetermined criteria [30]. The findings of this review synthesize the evidence examining further development, from supportive supervision to mentorship. Mentorship integrates elements that may be more successful in improving the quality of care through an individual focus on the professional, their goals and individual learning needs. A training and development model that is more empowering for the learner may also be more sustainable and support a decentralised model of primary health care that is predominant in developing countries.

### Further research

This review identified limited research investigating mentorship as a training and development strategy to improve the quality of care in developing countries. In order to improve quality and safety in LMICs, it is vital to conduct more research to clearly evaluate the effectiveness of specific models of mentorship in particular contexts and to determine what type of learning in healthcare benefits most from mentorship rather than supervision and in what contexts. While the literature favours supervision, a move towards a more power-shared model warrants further research and evaluation. Further studies could also investigate the appropriateness of health personnel from HICs mentoring health personnel from LMICs and further test desirable features of mentorship interventions as outlined in this review.

### Limitations

The scoping review methodology is appropriate when evidence is scarce and aligns well to the research aim which was to map existing evidence and identify research gaps [17]. Whilst the evidence was limited it did provide valuable insights into the characteristics and considerations of mentorship interventions in LMIC settings. Although attempts were made to contact authors, some challenges were encountered during study selection due to a lack of detail identifying whether the intervention involved mentorship. The terms supervision and mentorship were used broadly and interchangeably in the literature. While the authors separated these concepts into mutually exclusive categories, in practice this may not be the case.

## Conclusion

This review highlights a paucity of research on mentorship as a health personnel training and development strategy in low resource contexts. While mentorship is integrated into training and development programs in HICs, there may be a preference for supervision in LMICs, possibly representing an opportunity for innovation. Of the studies conducted to date, desirable features of successful mentorship interventions include matching the cultural background and discipline of mentors to mentees, providing appropriate training to mentors, and ensuring optimal intensity of the intervention given available resources. Other desirable features include integrating a relationship focused and supportive ‘side by side’ model of mentorship and ensuring mentorship programs are ongoing.

## Abbreviations

HIC: High-Income Country
HRH: Human Resources for Health
LMIC: Low and Middle-Income Country
